# Design, Optimization and Application of Small Molecule Biosensor in Metabolic Engineering

**DOI:** 10.3389/fmicb.2017.02012

**Published:** 2017-10-17

**Authors:** Yang Liu, Ye Liu, Meng Wang

**Affiliations:** Tianjin Institute of Industrial Biotechnology, Chinese Academy of Sciences, Tianjin, China

**Keywords:** metabolic engineering, synthetic biology, small molecule biosensor, transcription factor, optimization strategy, industrial application

## Abstract

The development of synthetic biology and metabolic engineering has painted a great future for the bio-based economy, including fuels, chemicals, and drugs produced from renewable feedstocks. With the rapid advance of genome-scale modeling, pathway assembling and genome engineering/editing, our ability to design and generate microbial cell factories with various phenotype becomes almost limitless. However, our lack of ability to measure and exert precise control over metabolite concentration related phenotypes becomes a bottleneck in metabolic engineering. Genetically encoded small molecule biosensors, which provide the means to couple metabolite concentration to measurable or actionable outputs, are highly promising solutions to the bottleneck. Here we review recent advances in the design, optimization and application of small molecule biosensor in metabolic engineering, with particular focus on optimization strategies for transcription factor (TF) based biosensors.

## Introduction

**FIGURE 1:**
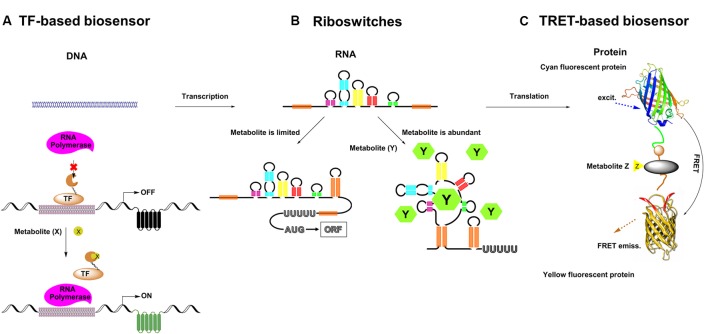
Three major categories of genetically encoded small molecule biosensors surrounding the central dogma. **(A)** Transcription factor (TF) based biosensor: the transcriptional inhibitory factor that can interact with its operator in the absence of its ligand (effector molecule) and dissociate from DNA when the ligand is abundant. TF activates expression of a reporter protein (e.g., GFP) in response to a target metabolite (‘X’); **(B)** Riboswitches: The RNA folds in local regions of complementarity, presumably, while transcription is proceeding. Cellular concentration of metabolites (‘Y’) under threshold concentration, transcription and intramolecular RNA folding are continue. Cellular concentration of metabolite (Y) above threshold concentration will be specifically sensed by sensor domains of riboswitches, and RNA folding can lead to an alternate conformation. **(C)** FÖrster resonance energy transfer (FRET) based biosensor: the binding of a metabolite (‘Z’) induces a FRET signal change.

**FIGURE 2:**
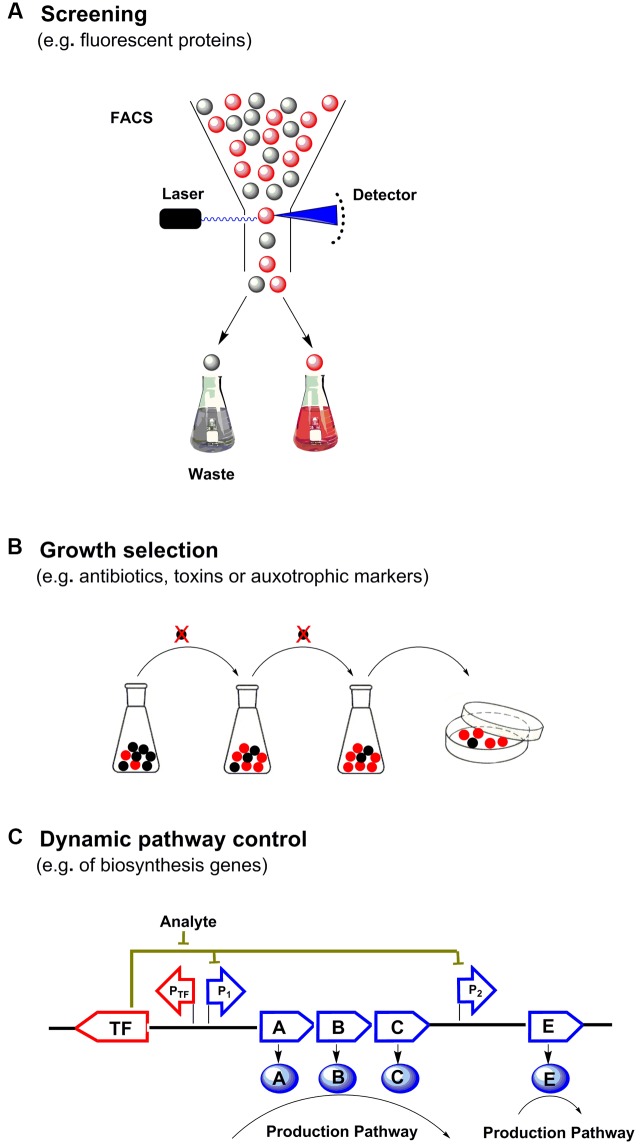
Applications of small molecule biosensors in metabolic engineering. **(A)** High throughput screening: the concentration of target metabolite is converted to fluorescence output at a single cell level, through fluorescence activated cell sorting (FACS) high-yield strains are enriched. **(B)** Growth selection: biosensors can be used to control the survival of strains with the desired traits by selecting suitable actuators (e.g., antibiotics, auxotrophs or toxins), which can directly enrich and select high-yield strains. **(C)** Dynamic pathway control: biosensors can further be used to build regulatory circuits to dynamically control and optimize the metabolic biosynthetic pathway.

Metabolic engineering has enabled the production of many chemicals currently produced from non-renewable resources using inexpensive and readily available raw materials (Keasling, 2010). In order to commercialize these processes, effective biosynthetic pathways must be built in suitable hosts, followed by extensive optimization to achieve economically feasible yields, titers and productivities (Liu D. et al., 2015). During the past decade, metabolite biosensors arise as one of the most powerful tools for metabolic engineering (Ng et al., 2015). In every living cell, a wide variety of metabolites are sensed by a broad range of natural sensors/actuators such as riboswitches, transcription factors (TF) or enzymes, and proper responses are carefully exerted by cell to maintain its function. In synthetic biology, researchers have borrowed, engineered or designed a series of sensors for specific small molecules. There are three major categories of genetically encoded small molecule biosensors surrounding the central dogma (**Figure 1**). And there are two major field of application for metabolite biosensors: (1) monitoring and screening the production of target products or important intermediates in engineered cells (Li et al., 2015; Wang et al., 2016). (2) establishing dynamic regulation network for biosynthetic pathways (Rogers et al., 2015; Rogers and Church, 2016) (**Figure 2**).

In the following sections, we review recent advances in the design, optimization and application of small molecule biosensor in metabolic engineering (**Table 1**), with particular focus on optimization strategies for TF based biosensors.

**Table 1:** Overview of three types of small molecule biosensors applied in metabolic engineering.

Type of biosensor	Analyte	Host chassis	Output	Sensor module	Reference
FRET	L-Leucine	*E. coli*	CFP + YFP	LivK of *E. coli*	Mohsin et al., 2013
FRET	L-Methionine	*S. cerevisiae*	CFP + YFP	MetN of *E. coli*	Mohsin and Ahmad, 2014
FRET	Glucose	*E. coli*	CFP + YFP	GGBP of *E. coli*	Fehr et al., 2003
FRET	Trehalose-6-phosphate	*E. coli*	eCFP + YFP	TreR of *E. coli*	Peroza et al., 2015
FRET	Pyruvate	Neurons	mTFP + Venus	PdhR of *E. coli*	San Martín et al., 2014
FRET	Lysine	*E. coli* and *S. cerevisiae*	CFP + YFP	LAO of *Salmonella enterica serovar typhimurium* LT2	Ameen et al., 2016
FRET	NADPH	*E. coli*	cpYFP	Rex of *Thermus aquaticus*	Tao et al., 2017
TF	L-lysine L-arginine L-histidine	*C. glutamicum*	eYFP	LysG of *C. glutamicum*	Binder et al., 2012
TF	L-valine	*C. glutamicum*	eYFP	Lrp of *C. glutamicum*	Mustafi et al., 2012
	L-leucine				
	L-isoleucine				
	L-methionine				
TF	Succinate	*E. coli*	TetA	DcuR of *E. coli*	Dietrich et al., 2012
TF	1-Butanol	*E. coli*	TetA-GFP	BmoR of *Thauera butanivorans*	Dietrich et al., 2012
TF	Triacetic acid lactone	*E. coli*	GFP	AraC of *E. coli*	Tang et al., 2013
TF	Malonyl-CoA	*E. coli*	eGFP	FapR of *B. subtilis*	Xu et al., 2013
TF	Lactams	*E. coli*	mCherry	ChnR of *Acinetobacter*	Zhang et al., 2017
TF	*p*-coumaric acid	*C. glutamicum*	YFP	PadR of *B. subtilis*	Siedler et al., 2017
TF	*S*-adenosylmethionine	*S. cerevisiae*	Venus	MetJ of *E. coli*	Umeyama et al., 2013
TF	NADH	*S. cerevisiae*	GFP	GPD2 of *S. cerevisiae*,	Knudsen et al., 2014
TF	NADPH	*E. coli*	eYFP	SoxR of *E. coli*	Siedler et al., 2013
TF	Xylose	*S. cerevisiae*	GFP	XylRs from *Bucillis*	Wang et al., 2016
TF	Malonyl-CoA	*S. cerevisiae*	tdTomato	FapR of *B. subtilis*	Li et al., 2015
TF	Benzoate	*E. coli*	GFP	BenR of *E. coli*	Uchiyama and Miyazaki, 2010
TF	Ectoine	*E. coli*	eGFP	AraC of *E. coli*	Chen et al., 2015
TF	L-threonine	*E. coli*	eGFP	a hybrid promoter of cysJp and cysHp	Liu Y.N. et al., 2015
Riboswitches	Coenzyme B12	*B. subtilis*	LacZ	5′-UTR of the *yvrC* gene from *B. subtilis*	Nahvi et al., 2004
Riboswitches	Glycine	*B. subtilis*	LacZ	5′-UTR of the *gcvT* operon from *B. subtilis*	Mandal et al., 2004
Riboswitches	Mg^2+^	*B. subtilis*	LacZ	the 5′-UTR of *mgtE* from *B. subtilis*	Dann et al., 2007
Riboswitches	Fluoride	*B. subtilis*	LacZ	*crcB* motif RNA from the *Bacillus cereus*	Baker et al., 2012
Riboswitches	Lysine	*C. glutamicum*	Citrate synthase (*gltA*)	5′UTR of the *lysC* gene coding from *B. subtilis*	Zhou and Zeng, 2015a

## Introduction to Genetically Encoded Small Molecule Biosensors

### FÖrster Resonance Energy Transfer (FRET) Based Biosensors

Genetically encoded FÖrster (or fluorescence) resonance energy transfer (FRET) based biosensors typically have a pair of donor and acceptor fluorophores (Zhang et al., 2015), which are usually fluorescent proteins (Zhang et al., 2002; Pakhomov and Martynov, 2008). The ligand binding domain is inserted between two fluorophores. When it is bound by the target ligand, the ligand binding domain induces a conformational change that altering the distance of the donor and receptor fluorophores, leading to a FRET signal change (Bermejo et al., 2011). The ligand-binding proteins could be periplasmic-binding proteins (PBP) (Fehr et al., 2003; Mohsin et al., 2013), regulatory proteins (Peroza et al., 2015), and other types of ligand-sensing protein. FRET-based biosensors have been used in the detection of a wide variety of small molecules, such as sugar phosphates (Peroza et al., 2015), pyruvate (San Martín et al., 2014), cofactors (Tang et al., 2014), amino acids (Kaper et al., 2007; Mohsin and Ahmad, 2014) and ions (Vinkenborg et al., 2009; De Michele et al., 2013; Ho and Frommer, 2014).

Trehalose-6-phosphate (T6P) plays a key role in sugar metabolism in bacteria, yeasts and plants. In order to directly visualize the concentration of intracellular T6P, Peroza et al. (2015) developed a series of specific T6P FRET sensors for *in vivo* microscopy. The FRET-based sensors were generated via fusion of the fluorescent proteins eCFP (enhanced cyan fluorescent protein) and Venus (yellow fluorescent protein) with trehalose repressor TreR from *Escherichia coli*. In addition to being directly involved in the metabolism of trehalose, T6P also regulates glucose metabolism and physiological processes as a signaling molecule. In order to test the utility of these biosensors *in vivo*, they examined the accumulation of T6P in *E. coli* cells under low- and high-osmolarity pressure. In low-osmolarity conditions, the addition of trehalose resulted in a FRET signal increase, indicating accumulation of T6P in cells. On the contrary, the signal was greatly reduced under high osmotic conditions, indicating that bypassing T6P, trehalose is directly converted to glucose. In the future, these biosensors can potentially be used for real-time monitoring of trehalose pathway regulations in any cell types (Peroza et al., 2015).

Amino acid industry, with annual production of more than 4 million tons, is a major pillar for industrial biotechnology. Lysine, one of the most demanding amino acids, has a global annual demand of about 1.5 million tones (Becker and Wittmann, 2012). Recently, Ameen reported a series of FRET-based biosensor for real-time measuring of intracellular lysine concentration. The reporter element LAO of the sensor is a lysine binding periplasmic protein, which was sandwiched between cyan fluorescent protein (CFP) and yellow fluorescent protein (YFP). In this work, the dynamic range for lysine detection was expanded by mutating amino acid residues that are critical for lysine binding. These engineered nanosensors can effectively monitor intracellular levels of lysine both in bacteria and yeast cells at different physiological scales (Ameen et al., 2016).

Most FRET-based biosensors are used to detect cytoplasmic small molecules and are rarely used in subcellular organelle such as mitochondria. In an interesting study, San Martín et al. (2014) made a FRET nanosensor for detecting pyruvate by flanking PdhR from *E. coli* with the fluorescent proteins mTFP and Venus. The application of this biosensor was performed in neurons, in which mitochondrial pyruvate consumption was discovered to go up by 300% within seconds by a strong calcium transient, while glucose levels remained unchanged (San Martín et al., 2014). Recently, Tao et al. (2017) developed and characterized a series of high performance genetically encoded fluorescent indicators for NADPH (iNap sensors). By properly designing and modifying ligand binding domains, they obtained sensors with different affinities and wide dynamic range. Using iNap biosensors, the research team accurately measured NADPH concentrations in cytosol and mitochondria of cancer cells and found that it was regulated by NAD kinase and glucose-6-phosphate dehydrogenase G6PD, demonstrating that NADPH metabolism in cancer cells is affected by glucose availability (Tao et al., 2017).

Although they are widely used, FRET-based biosensors have some shortcomings. Especially with its weak signal intensity, expensive and high sensitivity equipment are usually required. However, in Kim’s study, they developed a sensitive, inexpensive, and portable analyzer which can be used for quantitative measurement of small molecules content in liquid samples. The portable hand-held FRET analyzer was successfully evaluated by measuring sucrose and maltose contents in food items. It is expected that further miniaturization of the equipment and improvement of FRET-based biosensors will help the field application of the hand-held analyzer in the absence of expensive equipment, especially outside of laboratories (Kim et al., 2015).

FRET-based biosensors have the advantages of high orthogonality, temporal resolution and ease of construction. However, they can only report the abundance of target metabolites, and cannot implement downstream regulation in response to the signal. In addition, it is necessary to adjust the “bait” in a time-consuming manner according to the input operating range to increase the relatively low dynamic range. For the above reasons, FRET-based sensors are currently used primarily for monitoring intracellular metabolic dynamics rather than activities screening.

### Transcription Factor Based Biosensors

Transcription factors are sensory proteins that control cell physiology at the transcription level. A significant portion of TFs has evolved to regulate gene expression according to metabolite concentration or environmental changes (Zhang et al., 2015). Metabolite-responsive TFs have been integrated into a variety of synthetic gene circuits capable of detecting antibiotics (Binder et al., 2012), amino acids (Binder et al., 2012; Mustafi et al., 2012), vitamins (Eggeling et al., 2016), succinate (Dietrich et al., 2012), sugars (Raman et al., 2014; Kim et al., 2015), butanol (Dietrich et al., 2012), triacetic acid lactone (Tang et al., 2013) and malonyl-CoA (Xu et al., 2013, 2014). We will review their design and optimization strategies in detail in Section “Design and Optimization of TF-Based Biosensor.”

Lactams are a kind of important chemical raw materials used in the manufacture of polyamides. Recently, Zhang and his colleagues developed a lactam biosensor based on the ChnR/Pb TF-promoter pair. ChnR was placed under the control of a constitutive promoter and low copy number plasmid was used to reduce the metabolic burden of plasmid replication and protein production. The reporter protein mCherry was expressed under the control of the *Acinetobacter sp. chnB* promoter (Pb). In order to test the performance of the biosensor in *E. coli*, the addition of exogenous butyrolactam, valerolactam, and caprolactam were carried out, respectively. Valerolactam and caprolactam induced 2.38 and 2.08 fold fluorescence increase compared to the uninduced baseline, while a moderate 1.47 fold of induction was detected for butyrolactam. The TF-based lactam biosensor has sufficient specificity to differentiate lactams with a wide dynamic range, which would be potentially helpful for the high-throughput screening of high-titer lactam producing strains (Zhang et al., 2017).

*p*-Coumaric acid is an important precursor for the production of natural products, such as flavonoids, lignans and stilbenes, either in plants or heterologous microbial hosts (Marienhagen and Bott, 2013). PadR, a *Bacillus subtilis* transcriptional repressor, is naturally responsive to *p*-coumaric acid. Previous studies tried to build a PadR-based *p*-coumaric acid biosensor in *E. coli*. However, the PadR expression level in *E. coli* was too high, which led to strong inhibition of its downstream promoter. Siedler and his colleagues optimized the dynamic range of the PadR-based *p*-coumaric acid biosensor by varying its ribosomal binding site. Furthermore, they demonstrated that picoliter droplet technology can be used to co-culture *E. coli* and yeast. And droplets containing yeast cells producing high amounts of extracellular *p*-coumaric acid can be rapidly sorted out by using *p*-coumaric acid biosensor containing *E. coli* as a whole cell biosensor. After two rounds of screening, 96% of the 24 enriched yeast strains produced *p*-coumaric acid with a very similar yield of 0.17 ± 0.05 mM, indicating this screening system can be used to enrich *p*-coumaric acid producing strain (Siedler et al., 2017). However, its resolution still need further enhancement for screening high producing strains.

*Saccharomyces cerevisiae*, as another important workhorse in metabolic engineering, is used as host strain for the construction of many novel metabolite biosensors. Umeyama et al. (2013) engineered an *S*-adenosylmethionine (SAM) biosensor in *S. cerevisiae* by the fusion of MetJ repressor of *E. coli* and transcriptional activator domain B42. The resulting hybrid activator will bind SAM, which then can interact with the operator (*metO)* inserted upstream of the TATA box of *CYC1* promoter, leading to over-expression of downstream reporter gene. They used fluorescent protein Venus as the reporter for identifying unknown genes that will affect intracellular SAM concentration. As a result, *GAL11* was discovered as a novel multicopy enhancer of SAM level. Overexpression of *GAL11* resulted in an approximately 3.3-fold improvement in the concentration of SAM (Umeyama et al., 2013).

Knudsen et al. (2014) constructed a TF-based sensor that responded to the NADH/NAD^+^ ratio in yeast. GPD2, a glycerol triphosphate dehydrogenase, is responsible for the balance of NADH and NAD^+^ in yeast. The reporter gene GFP was expressed under the control of the GPD2 promoter. When the intracellular NADH was low, the promoter was activated to produce GFP. The sensor was the first reported biosensor that can detect NADH concentrations. Similarly, Siedler et al. (2013) developed an NADPH/NADP^+^ redox sensor in *E. coli* using its native redox sensitive TF SoxR. This biosensor was successfully used for the screening of NADPH-dependent alcohol dehydrogenase from *Lactobacillus aureus* (*Lb*Adh) mutants by fluorescence activated cell sorting (FACS). The activity of the enzyme mutant toward the substrate 4-methyl-2-pentanone was improved by 36% with an 8-fold increase in the *K*m value.

A large number of metabolite-responsive TFs exist in the cell, providing a rich source for engineering TF-based biosensors. And they have become the most widely used high-throughput screening tool in metabolic engineering. However, there are some key bottlenecks in construction and application of TF-based biosensor. It is still very challenging to transplant heterologous TFs into new hosts to generate functional biosensors. And detecting extracellular target metabolite concentrations seems out of the scope of TF-based biosensors.

### Riboswitches

Riboswitches are regulatory regions of mRNA molecules with conserved ligand-binding (sensor) domains and a variable sequence that can regulate downstream gene translation (Serganov and Nudler, 2013). Compared with TF-based sensors, riboswitches provide faster responses on account of the RNA has already been transcribed and thus can be immediately bound by effector and perform its regulation function. Riboswitches have been developed for the detection of purines and their derivatives (Vitreschak et al., 2004; Gilbert et al., 2006), coenzymes and related compounds (Nahvi et al., 2004; Serganov et al., 2009), amino acids (Mandal et al., 2004; Serganov et al., 2008), phosphorylated sugar (Klein et al., 2006) and antibiotics (Klauser et al., 2015). Some riboswitches specifically respond to inorganic ligands, including metal (Mg^2+^ cations) (Cromie et al., 2006; Dann et al., 2007) and fluoride anions (Baker et al., 2012).

The guanidine moiety is part of many important metabolites, including amino acid arginine, nucleobase guanine and energy carrier creatine (Nelson et al., 2017). Recently, Breaker et al. (2017) have experimentally validated the existence of three novel riboswitch classes that naturally sense free guanidine. The *ykkC* motif RNA, which is the longest riboswitch candidate, exists in much of the bacterial domain of life. Breaker et al. (2017) revealed that *ykkC* motif RNA commonly controls the expression of proteins may function as guanidine carboxylase and guanidine transporter, which were previously incorrectly annotated as urea carboxylases and multidrug efflux pumps (Nelson et al., 2017). The guanidine riboswitches control the expression of degradation or export proteins for detoxifying free guanidine.

Zhou and Zeng first reported using an amino acid riboswitch to improve the lysine biosynthesis. They used the natural lysine-OFF riboswitches of *E. coli* (ECRS) and *Bacillus subtilis* (BSRS) to regulate the expression of citrate synthase (*gltA*) gene and TCA cycle activity in *Corynebacterium glutamicum* LP917, respectively. The lysine production of the engineered strain ECRS-*gltA* and BSRS-*gltA* was 63% and 38% higher than their parent strain (Zhou and Zeng, 2015a). Furthermore, the natural lysine-OFF riboswitch was engineered to a synthetic lysine-ON riboswitch in their subsequent work. A lysine-ON riboswitch library was constructed and screened using *tetA*-based dual genetic selection system. Then a selected lysine-ON riboswitch was integrated into the *C. glutamicum* chromosome to regulate the expression of *lysE* gene (encoding a lysine transport protein). A 89% increase in lysine yield was achieved comparing to that of the *C. glutamicum* LPECRS strain with deregulated aspartokinase (Zhou and Zeng, 2015b). Their works provided a convenient and powerful tool for systematic and dynamic control of lysine production.

Although riboswitches are powerful tools, their discovery has met with challenges. First, many conserved mRNA elements were reported as candidate riboswitches without known natural ligands. For instance, the “*ykkC* motif” was reported as a candidate riboswitch in 2004, which was not until 2017 that its ligand was identified and named class I, or “guanidine-I,” riboswitches (Nelson et al., 2017). Second, currently, we do not have a rich history of research on the relationship between the structure of riboswitches and their corresponding metabolites. Serganov and Nudler (2013) pointed out that current biochemical and structural information is not enough for accurately predicting the ligand of every candidate riboswitch.

## Design and Optimization of TF-Based Biosensor

Although their designs are simple, the natural TF-based biosensor may have poor orthogonality and background noise resulting from the undefined interaction between TFs and operators in the native promoter (Lefrançois et al., 2009; Becker et al., 2015). Poor dynamic range and sensitivity of natural TF-based sensor is another concern in terms of applications in non-native conditions. These challenges can be addressed by introducing heterogeneous metabolite-responsive TFs from organisms known to respond to target metabolite. A few optimization steps are usually required for better performance in various applications. Here we reviewed most commonly used strategies for TF-based biosensor design and optimization

### Increase the Dynamic Range and Sensitivity

Although biosensor devices for small molecule detection are gaining increasing attention, very limited TFs have been successfully used in metabolic engineering. One of the majors challenges is their poor sensitivity and dynamic range (Liu et al., 2017). In a recent study, Liu et al. (2017) created a synthetic TF-promoter pair biosensor based on the native regulatory protein TyrR to monitor the variation of intracellular L-Phe concentration in *E. coli*. A series of optimization in the promoter region, including the insertion of extra nucleotides in the strong and weak promoter box, introducing a single mutation in the -10 region, and alteration of nucleotides in the discriminator sequence, were carried out. By optimizing the TF-promoter pair, TyrR showed a stronger activation effect, making the sensor better sensitivity and wider dynamic range. The dynamic range of the optimal biosensor was nearly 15 times higher than the original one, while its detection threshold is 10 times lower. When the biosensor is used as a FACS screening tool, the positive rate is increased from 86.2% to 99.5%, indicating that the optimized sensor is a more sensitive biosensor of L-Phe. Using this biosensor in a high throughput screening (HTS) platform, Liu et al. (2017) obtained a high L-Phe producing strain with a yield of 9.28 g/L.

In order to increase the dynamic range of a FadR-based biosensor for sensing fatty acid in *E. coli*, Zhang et al. (2012) introduced two copies of the operators into the strong phage lambda (PL) and phage T7 promoters (PA1). The dynamic range of the sensor increased by about 1000-fold. They also demonstrated that the dynamic sensor-regulator system significantly strengthened the stability of the biodiesel producing strain and enhanced the titer to 1.5 g/L. The yield has also increased three times to 28% of the theoretical yield (Zhang et al., 2012).

### Change Metabolite-Binding Specificity

Although the native TFs are capable of responding to many different kinds of metabolites, many target metabolites which produced by heterologous pathways may not be directly sensed by any native TFs or any TFs discovered yet. This challenge can be tackled by engineering metabolite specificity of known TFs. For instance, efforts have been made to modify AraC from the *E. coli ara* operon to sense D-arabinose (Tang et al., 2008), ectoine (Chen et al., 2015), triacetic acid lactone (Frei et al., 2016) and mevalonate (Tang and Cirino, 2011), instead of its native effector L-arabinose. For example, Tang et al. (2008) reported adopting saturation mutagenesis and FACS-mediated dual screening to isolate AraC variants which can’t bind with L-arabinose but specifically respond to D-arabinose.

Previous studies have shown that QacR, a tetR family repressor protein, can be induced by different compounds (Grkovic et al., 2003). In an interesting work (de los Santos et al., 2015), variants of QacR were produced through rational design and screened in a cell-free transcriptional translational system for novel response to vanillin, the most widely used flavor additive in food industry. It can potentially be used for screening a high vanillin producing strain in metabolic engineering works.

### Enable *de Novo* Design of Biosensors in Heterologous Hosts

One of the major advantages of synthetic biology is its ability to transfer functional parts between different organisms or even domains of life by following specific sets of engineering rules and processes. Because of its simplicity and abundance, prokaryotic metabolite-responsive TFs is a rich resource for constructing synthetic small molecule biosensors. On the contrary, eukaryotic small molecule sensory/regulatory mechanism usually involves multiple layers of signal transduction pathways. Despite its elegancy, it is not suitable for constructing metabolite sensor/gene circuit at this rudimentary stage of synthetic biology. Although it is still challenging, researchers have some success in transferring metabolite sensor between different domains of life. Stanton et al. (2014) developed a platform that enables the transfer of these regulators to mammalian cells. The 2,4-diacetylphloroglucinol (DAPG) sensing repressor PhlF of *E. coli* was fused with a nuclear localization signals (NSL) and the synthetic promoter was modified by incorporating six upstream operators. Therefore, the PhlF can repress downstream gene expression by steric hindrance. By introducing the DAPG biosensor, the new system will respond to extracellular DAPG with 50-fold induction ratio and 0.9 μM DAPG detection limit, which is comparable to the classic Dox-induced TetR system (Stanton et al., 2014). These examples show that it is feasible to design novel biosensors using elements from various species.

According to current genome annotation, there are more than 230 kinds of TFs in *E. coli*, which provide a vast toolkit for designing biosensors to sense different types of metabolites (Binder et al., 2012) in other microorganism. Indeed, early study has shown that bacterial transcriptional repressors can work in yeast (Brent and Ptashne, 1984). However, the rapid advancement of synthetic biology has made the design process more easy and reliable. Wang *et al.* engineered a novel xylose-sensing/regulation gene circuits in *S. cerevisiae* using a xylose-responsive TF XylRs from *Bucillis* and an engineered eukaryotic promoter GPM1p. In order to assess the effect of different hybrid promoters, they tried to insert the operator in three positions individually. And they found that while the downstream of the TATA-box and UAS were viable location for operator insertion, the best sensor dynamic range can be obtained by inserting operator just 1bp upstream of TATA-box. When the biosensor was used as a tool in directed evolution, they improved the xylose transport capacity of a sugar transporter HXT14 by 6.5-fold (Wang et al., 2016).

Following the same design rules, Li et al. (2015) reported the use of *Bacillus subtilis* trans-regulatory protein FapR and its corresponding operator *fapO* to design a malonyl-CoA sensor in *S. cerevisiae*. It is capable of reporting intracellular concentration of malonyl-CoA. By screening genomic overexpression library using the malonyl-CoA biosensor, an engineered yeast strain with increased malonyl-CoA concentration was obtained. And therefore, the titer of 3-hydroxypropionic acid was increased by 120% (Li et al., 2015). Such biosensors provide a powerful tool for genome-wide screening and can further improve the synthesis of a large number of chemicals derived from malonyl-CoA in yeast. Malonyl-CoA biosensor based on FapR-*fapO* system has also been used to control fatty acid production in *E. coli* (Xu et al., 2013, 2014). Xu et al. (2014) engineered a metabolic switch that can dynamically regulate the malonyl-CoA biosynthesis pathway (ACC) and the malonyl-CoA utilization pathway (FAS). The fatty acid production was improved greater than threefold in the strains with FapR regulating both ACC and FAS pathways.

## Industrial Application of Biosensors

### Used in the Screening of Industrial Enzymes

Environmental microbes is an excellent source of industrial enzymes (Ferrer et al., 2009). However, most environmental microbes cannot be cultured under laboratory conditions (Amann et al., 1995), which hampers our effort to obtain desired enzymes. One way to overcome this difficulty is to use biosensors as a tool to screen key enzymes with desired activities in the metagenomic library (Uchiyama et al., 2005). For instance, Uchiyama and Miyazaki (2010) developed a sensor that used a benzoate-responsive TF *benR* to activate the *benA* promoter (P*_benA_*) leading to GFP expression. The fluoresce intensity of *E. coli* cells carrying the biosensor would change in accordance with benzoate concentrations, but were completely unresponsive to the substrate benzamide. They used this benzoate biosensor to screen benzamidases from metagenomic library in *E. coli* and successfully obtained 11 different amidases from more than 96,000 clones.

Under industrial settings, wild-type enzymes often have limitations in different aspects such as activity and thermostability *etc*. Directed evolution is a powerful strategy for artificially improving enzyme properties in a short period of time. However, how to obtain a mutant enzyme with desired property from a mutant library usually with a significant size is a key challenge in directed evolution. A small molecule biosensor is a perfect solution for setting up a screening system in directed evolution experiments. For instance, Wu et al. (2017) obtained a LacI-L5 mutant that specifically responds to lactulose via screening of a saturation mutagenesis library of LacI. They applied the whole-cell lactulose biosensor in the directed evolution of cellobiose 2-epimerase (C2E) from *Caldicellulosiruptor saccharolyticus* for improved lactulose production. The selected mutant C2E enzyme was discovered to have ∼32-fold higher expression levels, which demonstrates that it is possible to use lactulose biosensor for screening lactulose high-producing strain in the future (Wu et al., 2017). In another example, Tang et al. (2013) engineered the *E. coli* TF AraC to induce gene expression by responding to intracellular triacetate lactone (TAL). Via high throughput screening, they obtained a highly active 2-pyrone synthase, with 20% increased activity.

### Used in the Screening of High-Yielding Microorganism

One of the main goals of metabolic engineering is to obtain a strain that can produce desired product with high yield. Many of the TF-based sensors targeting final products are used for high-throughput screening of high-yield microorganism. Ectoine is a compatible solute widely produced by bacteria for adapting to changes in osmotic stress (Lentzen and Schwarz, 2006). In the modern world, ectoine is often used in the cosmetic industry because of its anti-aging and skin-care properties (Graf et al., 2008). Because there is no known natural TF that can respond to ectoine, Chen et al. (2015) constructed a synthetic biosensor capable of sensing ectoine in *E. coli*. The authors changed the ligand specificity of AraC by changing the hydrophobic region of AraC, and obtained mutant AraC which can respond to ectoine. As a result of following ectonine biosensor based screening, the yield of ectoine was significantly improved by about 3.3-fold comparing to the wild-type ectoine production strain.

Biofuel is another major class of target chemicals that is actively pursued in metabolic engineering efforts. Many different biosensors were developed to sense biofuel molecules. Dietrich et al. (2012) constructed a biosensor capable of responding to 1-butanol in *E. coli* using the TF BmoR from *Pseudomonas butanovora* and its induced promoter P_BMO_. By combining the expression of *tetA* and *gfp* gene, the concentration of 1-butanol in the cell was coupled with the cell growth and fluorescence intensity. After a single round of positive selection, the 1-butanol production phenotype was improved by120-fold.

L-threonine is an important industrial amino acid, mainly used in animal feed industry, with annual production of 300,000 tons in 2014. And its market growth was expected to be over 20% in the next 5–10 years (Liu Y.N. et al., 2015). Although current threonine industrial fermentation technology achieves a high production level, there is still much room for further improvement. Liu Y.N. et al. (2015) developed a novel threonine sensor capable of sensing threonine in *E. coli* for setting up a high throughput screening system. The biosensor used a hybrid promoter of cysJp and cysHp and an eGFP as a reporter. Using FACS, over 400 strains were obtained from the library of 20 million mutants. 34 mutants were more productive than the initial industrial strains. In a 5 L fermenter, the best mutant was able to produce 17.95% more threonine.

Recent progress has also been made in the application of several natural and synthetic riboswitches in food, pharmaceutical and chemical industries. Naringenin, one of the plant flavonoids, has attracted tremendous interest due to its antioxidant function and human health related properties. Although there are two natural naringenin-responsive TFs (FdeR from *Herbaspirillum seropedicae* and TtgR from *Pseudomonas putida*) (Teran et al., 2003; Marin et al., 2013), Xiu et al. (2017) developed a riboswitch-based fluorescence biosensor using a naringenin-responsive RNA aptamer. The biosensor was designed to quantify the production of naringenin using a novel producer-biosensor co-culture system and can provide high-throughput rapid screening for naringenin high producers. In a recent study, an *N*-acetylneuraminic acid (NeuAc) biosensor was constructed based on a NeuAc-specific aptamer and a sfGFP fluorescence reporter in order to increase the yield of NeuAc, which is popular for its promising edible and pharmaceutical applications. With the designed biosensor, the NeuAc biosynthesis pathway was optimized by RBS library screening and key enzyme evolution, which improved the titer by 34% and 23%, respectively. Finally, a mutant with 8.31 g/L NeuAc production was obtained after two-stage fermentation in minimal medium with the glucose as sole carbon source (Yang et al., 2017).

## Conclusion and Future Perspectives

With the continuous advancement of biotechnology, more and more petroleum-based chemicals, fuels as well as pharmaceuticals can be produced through microorganisms from biomass even directly from CO_2_. Improving yield and production efficiency via metabolic engineering is a key step for their real industrial applications. Small molecule responsive biosensors have become and will continuously be one of the most powerful tools in real-time monitoring and systematic optimization of biosynthetic pathways in the metabolic engineering works.

In this review, we have examined the recent developments in metabolite-responsive biosensors and their various applications. Although there are many successful examples of metabolite-responsive biosensors from all three major categories, which were used in either the “proof-of-concept” experiments or achieved real impact in the industrial scale applications, there are some challenges need to be solved in a systematic way. First is how to streamline the discovery, design and optimization process of novel biosensors. It is very likely that every single natural metabolite has its own corresponding biosensor somewhere in the nature. However, how to quickly find them through enormous accumulated bioinformatics data and properly incorporated into a desired host via rational design is a major challenge. Second, despite that metabolite-responsive biosensors are extremely useful as a simple reporter/screening system, they will increasingly be incorporated into cellular control networks as a key layer that bridges small molecule and macromolecule. And how to design and optimize multiple metabolite-responsive biosensors that are compatible with each other and other genetic parts/devices is another grand challenge.

## Author Contributions

All authors listed have made a substantial, direct and intellectual contribution to the work, and approved it for publication.

## Conflict of Interest Statement

The authors declare that the research was conducted in the absence of any commercial or financial relationships that could be construed as a potential conflict of interest.
